# High-performance terahertz modulators induced by substrate field in Te-based all-2D heterojunctions

**DOI:** 10.1038/s41377-024-01393-6

**Published:** 2024-03-05

**Authors:** Pujing Zhang, Qihang Liang, Qingli Zhou, Jinyu Chen, Menglei Li, Yuwang Deng, Wanlin Liang, Liangliang Zhang, Qinghua Zhang, Lin Gu, Chen Ge, Kui-juan Jin, Cunlin Zhang, Guozhen Yang

**Affiliations:** 1 Department of Physics, Key Laboratory of Terahertz Optoelectronics, Ministry of Education, and Beijing Advanced Innovation Center for Imaging Theory and Technology, Capital Normal University, Beijing, 100048 China; 2 Beijing National Laboratory for Condensed Matter Physics, Institute of Physics, Chinese Academy of Sciences, Beijing, 100190 China; 3 Department of Materials Science and Engineering, Beijing National Center for Electron Microscopy and Laboratory of Advanced Materials, Tsinghua University, Beijing, 100084 China

**Keywords:** Terahertz optics, Optical materials and structures

## Abstract

High-performance active terahertz modulators as the indispensable core components are of great importance for the next generation communication technology. However, they currently suffer from the tradeoff between modulation depth and speed. Here, we introduce two-dimensional (2D) tellurium (Te) nanofilms with the unique structure as a new class of optically controlled terahertz modulators and demonstrate their integrated heterojunctions can successfully improve the device performances to the optimal and applicable levels among the existing all-2D broadband modulators. Further photoresponse measurements confirm the significant impact of the stacking order. We first clarify the direction of the substrate-induced electric field through first-principles calculations and uncover the unusual interaction mechanism in the photoexcited carrier dynamics associated with the charge transfer and interlayer exciton recombination. This advances the fundamental and applicative research of Te nanomaterials in high-performance terahertz optoelectronics.

## Abstract (web summary)

We have introduced Te-based all-2D heterojunctions as optically controlled terahertz modulators to significantly promote the device performances and elaborated their charge dynamics driven by substrate effect through first-principles calculations.

## Graphical abstract

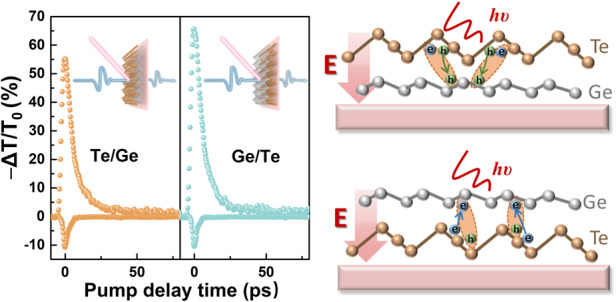

## Introduction

Terahertz (THz) technology has been considered as a potential candidate for the next generation communication system^1–3^. Wherein, high-performance modulator is one of the most significant components to load the information to the THz wave directly. However, the lack of suitable materials and effective active regulation has limited the development of this technology^4,5^. Two-dimensional (2D) materials with unique physical properties such as strong light-matter interactions, atomically thin profile, and fast carrier recombination, could offer an intriguing platform for investigating optoelectronic devices in fundamental physics^6–8^. Recently, the THz modulators based on 2D materials have attracted much attention with optical, magnetic, and electric control. Wherein, light field regulation provides higher degree of freedom and an easy access to tune the THz waves due to its rapid response and non-destructive contact^9–13^. At present, all-optically controlled THz modulator is faced with the tradeoff between modulation depth and speed, as well as the problem of insertion losses and bandwidth. Conventional semiconductor materials have high modulation depth but relatively slow modulation speed, which affects their development in ultrafast devices^14,15^. Although 2D materials have the transient response characteristics, the existing low modulation depth or the requirement of high pump fluence will hinder their practical applications^8,16,17^. Therefore, it is urgent to find out the favorable 2D materials to boost the device performances. The emerging mono-elemental 2D tellurium (Te) brings the dawn to this issue. This material with unique helical chain structure is of great superiorities, such as layer-dependent bandgap, extraordinarily high carrier mobility, strong optical response, and good air-stability^18–24^.

In addition, the heterojunctions fabricated by easily stacking different 2D materials can break through the limitation of lattice matching, which is required in the bulk semiconductor heterojunctions^25–27^. In particular, the dangling-bond-free surfaces of 2D layered materials enable the formation of an atomically sharp interface with on-demand properties^28–30^. Furthermore, the type­II band alignment is conducive for the separation and transfer of the photocarriers^31,32^. This is crucial for the light-electric interconversion at the van der Waals (vdW) heterointerface in the optoelectronic applications. For example, the heterojunction of 2D Te and transition metal dichalcogenide can construct the highly-efficient solar cells^33^. Other infrared photodetectors using Te-based vdW heterojunctions achieved a high detectivity and fast response time^23,32^. However, few studies have been carried out on the high-performance THz modulators integrated with 2D Te heterojunctions. It is expected that such THz modulators can be regulated through heterojunction interface to provide an exploratory possibility for functional devices. Importantly, as a powerful technique to measure complex photoconductivity after optical excitation, THz wave can also probe the dynamics of free carriers or bounded excitons in a nondestructive way^9,34^. Our investigation of THz photodevices formed by all-2D Te-based heterojunctions could reveal the vdW interlayer coupling and the carrier dynamics. It is helpful to further understand the relationship between the heterointerface formation and substrate effect, facilitate the multiparameter optimization of the modulation performance, and clarify the working principle of the novel THz devices.

Here, combined with substrate engineering, we have first proposed an efficient strategy to introduce Te-based nanofilms as a new class of optically controlled THz modulators. It is found that the low-loss and broadband Te nanofilms can achieve high modulation depth in a picosecond timescale and show an ultrasensitive response under low pump excitation. Further parameter optimization can be realized in their formed vdW heterojunctions. Especially, Ge/Te exhibits an enhanced modulation depth of 87.6% along with short-lived dynamics of less than 8 ps relaxation time that originates from the charge transfer and interlayer exciton recombination. Moreover, the measurements display the ohmic-like contact in Te/Ge but good rectifying behavior in Ge/Te, implying the stacking order can significantly modify the energy band structure induced by the substrate effect. By calculating the space distribution of the differential charge density with density functional theory (DFT), we first validate the existence of the substrate-induced electric field and clarify its influence on the charge transfer process of non-equilibrium state after photoexcitation. Our obtained results show the Te-based all-2D vdW heterojunctions with the substrate engineering can improve the device performances remarkably and open up a new idea for the design, optimization, and application of the optically controlled highly-efficient THz modulators.

## Results

### THz transient dynamic process of devices

**Fig. 1:**
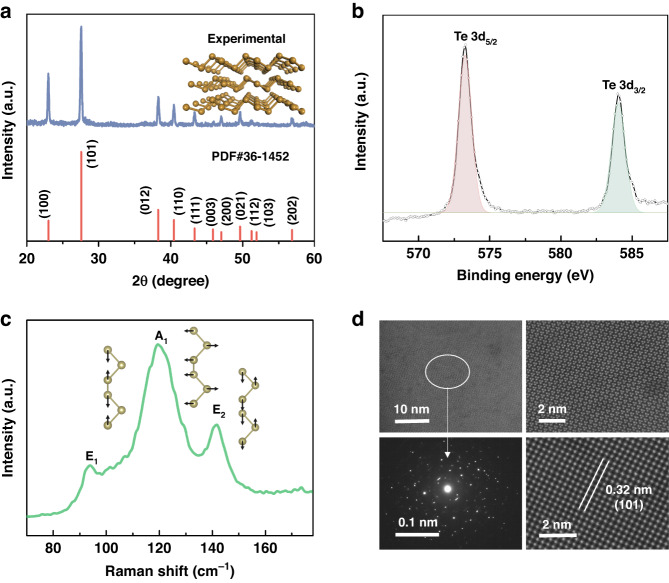
Characterization of Te nanofilms. **a** XRD patterns. **b** XPS spectrum. **c** Raman spectrum. **d** HRTEM images. The measured lattice distance is 0.32 nm, corresponding to the (101) planes of Te crystal

High-quality and large-area Te nanofilms were deposited by the electron beam evaporation method on the fused silica substrates. The X-ray diffraction (XRD) pattern to examine the structure and crystalline nature is shown in Fig. 1a. Typical characteristic diffraction peaks of (101) and (110) for Te (PDF card no. 36-1452) can be detected. As presented in Fig. 1b, X-ray photoelectron spectroscopy (XPS) spectrum of Te nanofilm exhibits two obvious peaks with the binding energies of 573.2 and 583.9 eV, which are assigned to Te 3d_5/2_ and Te 3d_3/2_, respectively. The Raman spectrum is shown in Fig. 1c. The prominent peaks located at 94, 121, and 142 cm^−1^ can be attributed to the *E*_1_, *A*_1_, and *E*_2_ vibration modes, which are caused by the asymmetric bond-stretching along *c*-axis and assigned to predominately bond-bending, chain expansion, and bond-stretching types, respectively^19–21,32^. To further confirm the crystal structure of different domains, high-resolution transmission electron microscopy was performed as displayed in Fig. 1d, which directly illustrates the crystalline state of Te layer and shows trigonal structure^20,22^. Other characterizations are presented in Fig. S1, showing the thickness is 100 nm with the optical bandgap of 0.37 ± 0.01 eV^35^.

**Fig. 2:**
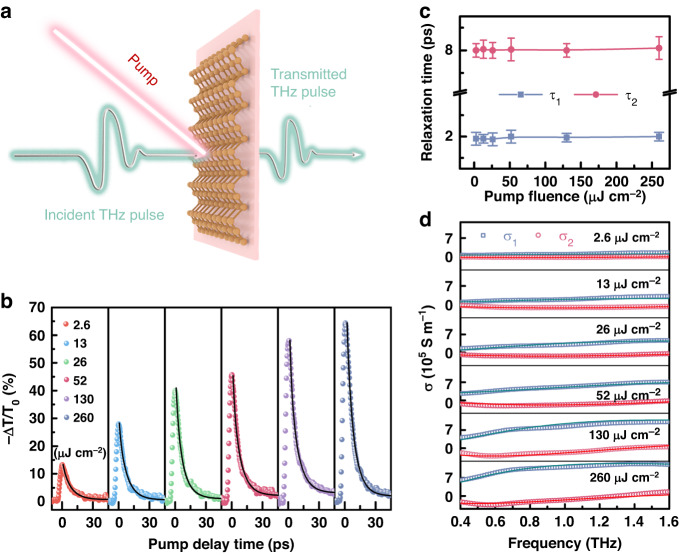
Transient dynamics properties of Te. **a** Schematic illustration of THz measurement scheme. **b** Transient THz dynamics of Te (100 nm) under different pump fluence with 800 nm excitation wavelength. Black solid lines represent the biexponential fitting curves. **c** Pump fluence dependence of the fitted relaxation times. **d** Measured complex photoconductivity spectra. Solid lines represent the Drude-Smith fitting curves

Our schematic experimental scheme is illustrated in Fig. 2a using optical pump-THz probe technology to investigate the active modulation response of Te-based THz devices^14^. The measured transient THz dynamics is demonstrated in Fig. 2b for Te (100 nm) with 800 nm pump excitation. The relative change is defined as −Δ*T*/*T*_0_ = − (*T* − *T*_0_) /*T*_0_, where *T*_0_ and *T* are the THz peak values before and after the photoexcitation. It is found that −Δ*T*/*T*_0_ first shows a rapid positive change and reaches its maximum within several picoseconds after the photoexcitation owing to the generation of hot carriers^36,37^. The subsequent relaxation processes under different pump fluence are well fitted by the biexponential functions, and the extracted relaxation times *τ*_1_ and *τ*_2_ are displayed in Fig. 2c (see Table S1 for details). It is shown that the decay consists of two stages with distinct timescales. The fast recovery occurs within 2 ps, which represents the energy relaxation of the carriers. This pump-independent trend of *τ*_1_ might originate from the electron-phonon scattering process^11^. Then a slow recovery lasts for about 8 ps to depict the carrier lifetime and its independence on pump fluence suggests that the synergistic interactions between Auger effect and defect trapping dominate the photocarrier dynamics of Te^38^. Therein, the recombination centers formed by impurities and defects promote recombination and shorten the carrier lifetime, while the formation of the trap center prolongs the lifetime. Under 400 nm pump excitation, the relaxation time constants remain unchanged (Supplementary Fig. S2). This feature is beneficial to accomplish the unperturbed modulation speed in the optical active devices since the carrier lifetime is usually deteriorated with the increased pump intensity. In addition, the transmitted THz wave can be significantly attenuated, which can be described by modulation depth of *MD* = | −Δ*T*/*T*_0_*|*_*max*_. It can be seen that the *MD* of Te (100 nm) is 14.5% even at an extremely low fluence of 2.6 µJ cm^−2^ and can reach 65.3% at 260 µJ cm^−2^. The corresponding transmission spectra at pump delay time of 0 ps exhibit the fluence-dependent but frequency-independent broadband features (Supplementary Fig. S3). It is worth noting that this material has a high transmission of 96.3% without pump, leading a very low insertion loss of −0.33 dB calculated by 20lg (*E*_sam_/*E*_sub_), where *E*_sam_ and *E*_sub_ are the THz amplitudes for sample and substrate, respectively. Different from the behaviors of Te (300 nm) with much longer carrier lifetime and Te (50 nm) with relatively low *MD* (Supplementary Fig. S4), Te (100 nm) is the optimal candidate to accomplish the excellent modulation characteristics. Furthermore, we calculate the skin depth of the pumped film at THz frequencies given in Fig. S4d, which confirms that the film can significantly suppress THz transmission. To better reveal the optical properties of Te (100 nm) in the THz region, we have calculated the complex photoconductivity with the real part *σ*_1_ and imaginary part *σ*_2_ displayed in Fig. 2d under different fluence at pump delay time of 0 ps. The modified Drude-Smith conductivity formula was derived with a more solid physical foundation and well-defined fit parameters^39^. This model, which is based on a diffusive restoring current to provide a direct connection between THz conductivity and microscopic particle motion, can be expressed by $\widetilde{{\rm{\sigma }}}\left(\omega \right)$ = $\frac{N{e}^{2}{\tau }^{{\prime} }}{m\left(1-i\omega {\tau }^{{\prime} }\right)}\left[1-\frac{1}{1-i\omega {t}_{d}}\right]$, where *N*, *e*, *m*, *ω, τ‘*, and *t*_*d*_ are charge carrier density, elementary charge, effective mass, plasma frequency, total scattering time, and diffusion time, respectively^39–41^. The fitted curves in Fig. 2d exhibit a good agreement with experimental data. The fitting parameters are given in Table S2. It is found that the carrier total scattering time (*τ‘*) reduces from 750 to 375 fs and diffusion time (*t*_*d*_) rises from 21 to 53 fs due to the enhancement of carrier concentration with the increased pump fluence^35,42,43^.

**Fig. 3:**
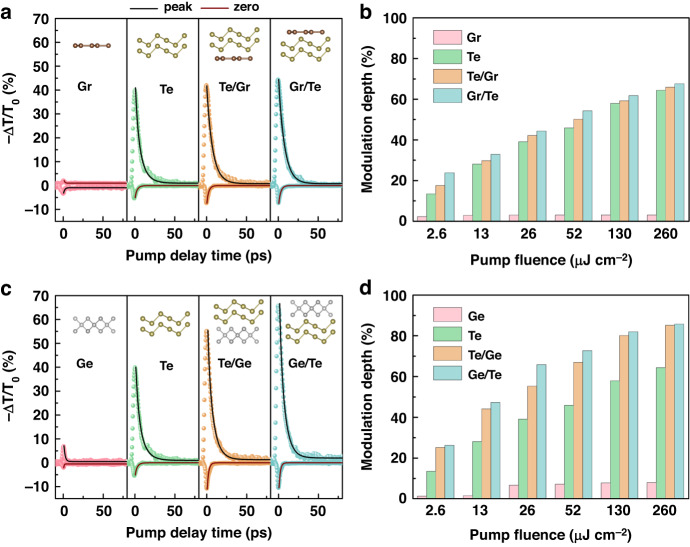
Transient dynamics properties of Te-based heterojunctions. **a** −Δ*T*/*T*_0_ at the peak and zero crossing of THz amplitude under 800 nm pump with fluence of 26 µJ cm^−2^ and (**b**) fluence-dependent modulation depths for Gr and Te, Te/Gr and Gr/Te heterojunctions, as well as (**c**, **d**) for Ge and Te, Te/Ge and Ge/Te heterojunctions, respectively

Since the heterointerface could enhance the photoelectric conversion efficiency, the Te-based heterojunctions are expected to further improve the performance of devices. Firstly, we have studied the THz responses of heterojunctions formed by Te (100 nm) and monolayer graphene (Gr) in different stacking orders. Figure 3a demonstrates −Δ*T*/*T*_0_ at the peak of THz amplitude for Gr and Te, Te/Gr and Gr/Te heterojunctions under 800 nm pump with the fixed fluence of 26 µJ cm^−2^. It is known that Gr exhibits negative THz photoconductivity, which is ascribed to the enhancement of carrier scattering rate overtaking the increase in Drude weight^6^. The obtained positive THz photoconductivities of Te, Te/Gr, and Gr/Te indicate the main contribution from the free carriers. Moreover, relaxation processes still have two distinct decay components. It is obvious that when Gr and Te are combined into the heterojunction, the transient response does not decrease but slightly increases, showing that the heterointerface plays an important role in the carrier dynamics. Here, the photon excitation creates free carriers, and these charged carriers will rapidly transfer between the two layers. The black solid lines are the biexponential fitting of −Δ*T*/*T*_0_ at the peak of THz amplitude. The extracted time constants are *τ*_1_ = 1.95 ± 0.12 ps and *τ*_2_ = 8.00 ± 0.25 ps in Te, *τ*_1_ = 1.85 ± 0.15 ps and *τ*_2_ = 7.80 ± 0.20 ps in Te/Gr, *τ*_1_ = 1.80 ± 0.15 ps and *τ*_2_ = 7.90 ± 0.25 ps in Gr/Te, respectively. The slight decrease of in relaxation times in two heterojunctions suggests the possibility of exciton existence^16,26,30^.

To further clarify the charge species and the transfer in the heterojunction, we can monitor the photoinduced dynamics of excitons since the THz photon allows it to couple with the intraexcitonic transition of bounded charge. In experiments, this can be quantified by scanning the change of the THz amplitude at a zero-crossing point (Supplementary Fig. S5), through which one is able to exclude the contribution of the photoinduced amplitude change^9,11,12,26^. The obtained transient curves presented in Fig. 3a depict the exciton dynamics fitted with red solid lines, displaying the exciton formation and annihilation via recombination to finally determine an exciton lifetime. We have found that the exciton lifetimes of Te, Te/Gr, and Gr/Te are significantly shorter than those of free carriers (Supplementary Table S3) due to the contribution from weak excitons. Especially, the lifetimes of both free carriers and excitons in the heterojunctions are further slightly reduced accompanied with the emergence of increased exciton component, indicating that electron-hole pairs are not only in the individual layers but also across the interface as the interlayer excitons. Thus, the picosecond carrier lifetimes are assigned to the charge transfer through the interface and subsequent interlayer exciton decay. The extracted *MD* under various fluence is presented in Fig. 3b. It can be seen with the increased pump fluence, the *MD* of Gr quickly reaches saturation with relatively low value of 2.8%. Compared with that of Te, the *MD*s of two heterojunctions are higher and exhibit larger difference at low fluence. Furthermore, the stacking order of Gr and Te has the obvious influence on the modulation property. It has been mentioned above that the *MD* of Te is 14.5% at pump fluence of 2.6 µJ cm^−2^ but Gr/Te can reach 23.5%, verifying the great potential of all-2D heterojunction to realize the modulation enhancement. At pump fluence of 260 µJ cm^−2^, the value of Gr/Te is 69.0% with little difference from that of Te, suggesting the limited enhancement with the use of Gr and Te at high fluence.

It is known that the optical properties of the heterojunction are closely related to the material component and its interface. As an indirect band gap semiconductor, Ge has considerable potentials and can form the heterostructure with Te to improve device performance. Hence, we have further studied the THz transient responses of all-2D vdW heterojunctions formed by Te (100 nm) and Ge (100 nm). Figure 3c shows −Δ*T*/*T*_0_ at the peak and zero crossing of THz amplitude for Ge, Te, Te/Ge, and Ge/Te at 800 nm with pump fluence of 26 µJ cm^−2^. The transient behavior of Ge/Gr and Gr/Ge is given in Fig. S6 for comparison. We can find that the optical excitation behavior is not significant in Ge with the negligible proportion of excitons. However, the photoexcited phenomena of Te/Ge and Ge/Te heterojunctions are more remarkable than those of not only individual component material but also the above Te/Gr and Gr/Te heterojunctions. The extracted time constants are *τ*_1_ = 1.60 ± 0.10 ps and *τ*_2_ = 7.70 ± 0.20 ps in Te/Ge, *τ*_1_ = 1.50 ± 0.10 ps and *τ*_2_ = 7.75 ± 0.25 ps in Ge/Te, respectively. Compared with the heterojunctions formed with Gr, those shorter relaxation times of free carriers and excitons (Supplementary Table S3) are the evidence of ultrafast and highly efficient charge transfer across the interface and the subsequent interlayer exciton decay. Moreover, the *MD* at 26 µJ cm^−2^ is significantly enhanced with the value of 55.4% for Te/Ge and 69.5% for Ge/Te, respectively. Figure 2d presents their *MD*s under different fluence. Wherein, the *MD* of Ge is also very small and insignificant because it only reaches 8.3% at high pump fluence. When Ge and Te are combined to form heterojunction, the *MD* increases considerably. Especially, the *MD* of our all-2D Ge/Te heterojunction can achieve 27.8% under the extremely low pump fluence of 2.6 µJ cm^−2^ and further accomplish an ultrahigh value of 87.6% at 260 µJ cm^−2^, which is one of the key results of this work compared with other reported 2D modulators, as shown in Table 1^13,26,27,36,38,44^. This implies that Ge/Te vdW heterojunction can effectively enhance the modulation depth while maintaining the fast speed, which may be due to the effect of stacking order on charge transfer and interlayer coupling.

**Table 1:** Comparison between our work and other 2D THz modulators

2D material	Tuning methods	Pump wavelength	Modulation speed	*MD* @ low fluence	*MD* @ high fluence
CdTe^13^	Optical	400 nm (3.1 eV)	ps	2.3% (29 µJ cm^−2^)	9.5% (174 µJ cm^−2^)
PtTe_2_^44^	Optical/Thermal	780 nm (1.59 eV)	ps	12% (60 µJ cm^−2^@5 K)	27% (362 µJ cm^−2^@5 K)
PdSe_2_^38^	Optical/Thermal	780 nm (1.59 eV)	ps	1.1% (72 µJ cm^−2^)	2.4% (192 µJ cm^−2^@100 K)
Cd_3_As_2_/CdTe^36^	Optical	800 nm (1.55 eV)	ps	12.3% (6.35 µJ cm^−2^)	80% (2500 µJ cm^−2^)
MoTe_2_/WTe_2_^26^	Optical	1550 nm (0.8 eV)	sub-ps	7.5% (10 µJ cm^−2^)	——
Graphene/PtSe_2_^27^	Optical	1300 nm (0.95 eV)	ps	0.5% (9 µJ cm^−2^)	2.2% (362 µJ cm^−2^)
Te (this work)	Optical	800 nm (1.55 eV)	ps	14.5% (2.6 µJ cm^−2^)	65.3% (260 µJ cm^−2^)
Ge/Te (this work)	Optical	800 nm (1.55 eV)	ps	27.8% (2.6 µJ cm^−2^)	87.6% (260 µJ cm^−2^)

### Photoresponse properties of devices

**Fig. 4:**
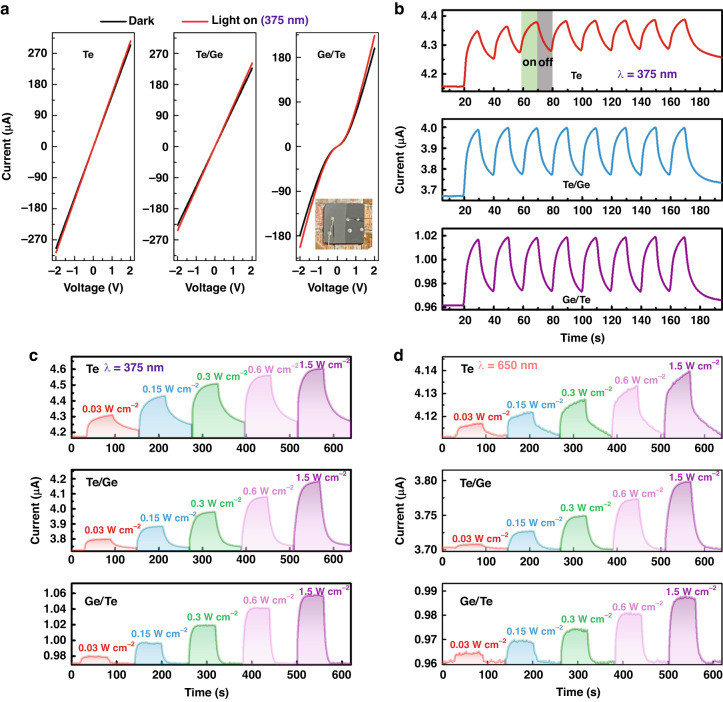
Photoresponse measurements of Te, Te/Ge and Ge/Te heterojunctions. **a** I*–*V curves of Te, Te/Ge and Ge/Te heterojunctions in dark and light conditions (375 nm 0.3 W cm^−2^), respectively. The inset shows the optical photo of Ge/Te heterostructure. **b** Time-resolved photoresponses of the samples under 375 nm with 0.3 W cm^−2^ (on/off = 10/10 s) at *V*_*bias*_ of 0.1 V. **c** Photo-switching characteristics measured at *V*_*bias*_ of 0.1 V under 375 nm and (**d**) 650 nm with different light powers (on/off = 60/60 s), respectively

To explore the physical mechanism of the stacking effect on the device performances, we measured the photoelectric responses to provide the interface information related with ultrafast behavior and modulation capability. The I–V curves of Te, Te/Ge and Ge/Te heterojunctions are presented in Fig. 4a under dark and light conditions. The linear relationship indicates the ohmic contact formed at their interface between Te nanofilm and Ag electrodes, as well as Ge and its electrodes (not shown). Notably, for the Te/Ge heterojunction, we also find the linear characteristic like ohmic contact, suggesting there is no obvious potential barrier at the formed Te/Ge heterointerface. However, I*–*V curve of Ge/Te is nonlinear with rectifying behavior, showing the emergence of barrier at their interface^19,32^. Figure 4b depicts their transient current response under 375 nm light irradiation. When the illumination is repeatedly turned on and off with on/off = 10/10 s, the presented eight cycles of ‘on-off’ state switching retain similar photocurrent, suggesting high stability and repeatability of device^24,32^. It can be found the photocurrent recoveries in two heterojunctions are more rapid, especially for Ge/Te. We further prolong the on/off time to 60/60 s to measure the photoresponse under the irradiation of 375 and 650 nm laser with different powers, as shown in Figs. 4c, d, respectively. It is also revealed that only the Ge/Te heterojunction can switch quickly between low and high current states with steep rise and fall edges, indicating that electron-hole pairs could be effectively transferred and recombined. This coincides with the efficient charge transfer and strong coupling between Ge and Te through the interface. Such dynamical response difference is of great importance for the design and fabrication of vdW heterostructures.

### Theoretical analysis of substrate-induced carrier dynamics

**Fig. 5:**
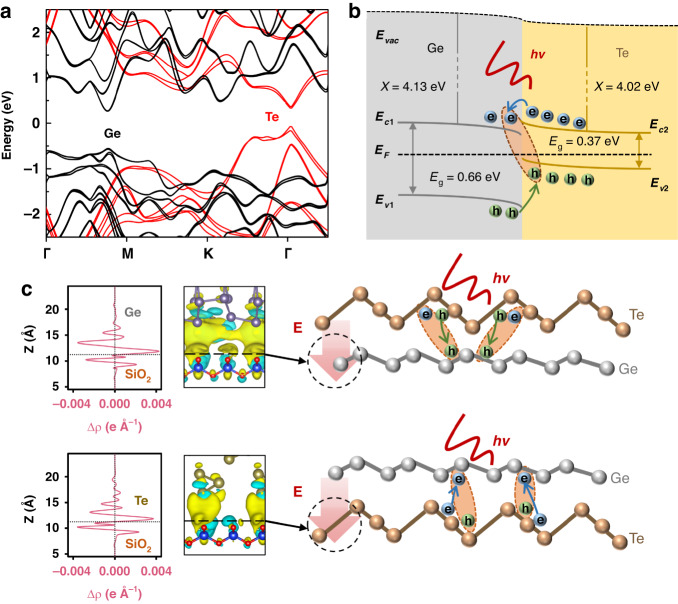
Substrate-induced carrier dynamics in Te/Ge and Ge/Te heterojunctions. **a** Calculated band structures and (**b**) Energy band diagram of Ge and Te. **c** Left: Calculated space distribution of the differential charge density for different materials on substrate. The color blue (yellow) denotes electron depletion (accumulation). Right: Schematic substrate-induced electric fields and charge transfer in heterojunctions of Te/Ge (upper) and Ge/Te (lower), respectively

Here, we assume that the effective field of the substrate engineers the band structure of the heterogeneous interface through the stacking order, and thus the optical transient behaviors can be regulated. According to the calculated electronic band structures of Ge and Te in Fig. 5a, we illustrate energy band diagrams of heterojunction in Fig. 5b (see calculation details in Supplementary Information). The electron affinity and bandgap are about 4.13 eV and 0.66 eV for Ge, and 4.02 eV and 0.37 eV for Te^19–21,45,46^. Due to the natural defects formed in the growth process, Te and Ge are usually weak *p*- and *n*-type semiconductors, respectively, with the Fermi levels near their individual intrinsic Fermi levels^19,21,46^. It can be seen that both the conduction band minimum (*E*_*c*_) and the valence band maximum (*E*_*v*_) of Te are higher than those of Ge with the type-II heterostructure. The photogenerated electrons in Te can be transferred to Ge layer through thermionic emission across the barrier. Meanwhile, the holes in Ge will move to Te. Hence, the interlayer exciton can be formed at the interface layer. When considering the effective electric field introduced by the substrate, the stacking order will cause the Fermi level shifting in Ge or Te layer. We have calculated the space distribution of the differential charge density between our used 2D materials and the fused silica substrate using DFT, as displayed in the left of Fig. 5c. The calculation results indicate that the direction of the effective electric field points to the substrate, leading to an electron accumulation layer at the side of the 2D material^47,48^. For Te/Ge, the substrate effect could shift the Fermi level of Ge upward to be close to that of Te. This will induce the reduction of the interface barrier to form the ohmic-like contact, which is consistent with the measured linear current in Fig. 4a. On the other hand, the substrate-induced effect can raise the Fermi level of Te, leading to the increased barrier at the interface of the Ge/Te heterojunction with prominent rectification characteristics. This can be also proved by the surface potential difference between Te and Ge measured by AFM (Supplementary Fig. S7)^32^.

Furthermore, the different optical modulation performances of those two heterostructures can be also described by the substrate field effect across the interfaces, as illustrated in the right of Fig. 5c. Here, we consider that the photogenerated carriers in Te play a dominant role after light excitation. For Te/Ge, the photoexcited holes can be transferred from the top Te to the bottom Ge under the effective electric field of the substrate. However, this process is extremely limited due to the large barrier in the valence band. For Ge/Te, the substrate induced electric field can significantly promote the transfer of photoexcited electrons from bottom Te to top Ge since their barrier in conduction band is relatively small with the permission of thermionic emission, thus increasing the THz photoconductivity. Simultaneously, the formation of interlayer excitons is promoted to shorten the carrier lifetime. It is noticed that this substrate-induced effect has a great impact on the charge transfer process under low pump photoexcitation^27^. Under high pump fluence, the excess photocarriers could partially screen the electric field, leading to the less influence of stacking order on modulation depth. Our obtained results indicate the substrate engineering can effectively improve the device performances in all-2D THz modulators.

## Discussion

In summary, in view of the tradeoff between optical modulation depth and speed in the THz modulators, we have first proposed an approach to use the novel Te nanofilms combined with the heterostructure to promote device performances. The pure film modulator with low insertion loss and broadband width can achieve high modulation at the picosecond timescale and exhibit the ultrasensitive optical response at low excitation fluence. It is found that the highly efficient charge transfer and the formation of interlayer weak excitons at the heterointerface can enhance the modulation with the maintained ultrafast behavior. In particular, the Ge/Te vdW heterojunction achieves an ultrahigh *MD* of 87.6% with the shortened relaxation time of less than 8 ps and suggests the great influence of stacking order. Further photoresponse experiments exhibit the obvious rectification effect in Ge/Te due to the interface barrier. The subsequent DFT calculation and analysis first clarify the substrate-induced field could engineer the band structure to support the thermionic emission and the formation of interlayer excitons, thereby improving the ultrafast modulation properties. Our obtained results could provide a more comprehensive understanding on the internal mechanism of ultrafast charge transfer and exciton dynamics in all-2D heterostructures, guide the design of vdW interfaces, and envision a new class of power-efficient, high speed, low insertion loss, and broadband tunable THz photonic devices.

## Materials and methods

### Sample preparation

Te nanofilms were grown on the fused silica substrates in an electron-beam evaporator (VZS 600 Pro) with an in situ thickness meter. Before the deposition, fused silica substrates were cleaned by ultrasonication in acetone for 10 min and rinsed with isopropyl alcohol and deionized water. The deposition of Te was initiated from a 99.999% pure Te source in a boron nitride crucible at a rate of 0.1 nm s^−1^ under 10^−4^ Pa pressure. Annealing was performed in a tube furnace at 373 K under a 100 sccm Ar atmosphere for 0.5 h. Monolayer Gr and Ge nanofilms from SixCarbon Technology (Shenzhen, China) were transferred onto the prepared samples.

### Material characterization

XRD patterns was performed using a Rigaku SmartLab instrument with a 2*θ* range from 20 to 80^o^ in step of 0.05^o^. XPS measurements were performed on ThermoFisher Scientific ESCALAB 250X under monochromatic Al Kα radiation with an energy of 1486.6 eV. Raman spectrum was analyzed using the alpha300 R microscope under 532 nm laser excitation. The surface morphology of 2D Te was measured using Dimension ICON atomic force microscope and FEI Sirion scanning electron microscope. Absorption spectrum was performed using the Cary7000 UV-VIS-NIR Spectrophotometer.

### Devices of photoresponse studies

Photoresponse were measured in a Lakeshore probe station with a Keithley 4200 semiconductor parameter analyzer in air at room temperature. The laser with a wavelength of 375 nm and 650 nm were used for the optical switching in the experiment.

### Supplementary information

Supplementary Information for High-Performance Terahertz Modulators Induced by Substrate Field in Te-Based All-2D Heterojunctions

## Data Availability

All relevant data are available within the Article and Supplementary Information, or available from the corresponding authors upon reasonable request.
