# Supplementary material for: High-performance terahertz modulators induced by substrate field in Te-based all-2D heterojunctions
Source: Light Sci Appl. 2024 Mar 5;13:67. doi: 10.1038/s41377-024-01393-6 (PMC10915160; doi:10.1038/s41377-024-01393-6)
Supplement: Supplementary file 1 — Supplementary Information for High-Performance Terahertz Modulators Induced by Substrate Field in Te-Based All-2D Heterojunctions [file 41377_2024_1393_MOESM1_ESM.docx]

**Supplementary Information for**

**High-Performance Terahertz Modulators Induced by Substrate Field in Te-Based All-2D Heterojunctions**

Pujing Zhang,^1^ Qihang Liang,^1^ Qingli Zhou,^1,*^ Jinyu Chen,^1^ Menglei Li,^1^ Yuwang Deng,^1^ Wanlin Liang,^1^ Liangliang Zhang,^1^ Qinghua Zhang,^2^ Lin Gu,^3^ Chen Ge,^2,*^ Kui-juan Jin,^2^ Cunlin Zhang,^1^ Guozhen Yang^2^

^1^ Key Laboratory of Terahertz Optoelectronics, Ministry of Education, and Beijing Advanced Innovation Center for Imaging Theory and Technology, Department of Physics, Capital Normal University, Beijing 100048, China

^2^ Beijing National Laboratory for Condensed Matter Physics, Institute of Physics, Chinese Academy of Sciences, Beijing 100190, China

^3^ Beijing National Center for Electron Microscopy and Laboratory of Advanced Materials, Department of Materials Science and Engineering, Tsinghua University, Beijing 100084, China

* Correspondence and requests for materials should be addressed to Q.Z. (email: qlzhou@cnu.edu.cn) or to C.G. (email: gechen@iphy.ac.cn).

**1. Sample characterizations**

The atomic force microscopy (AFM) image as presented Fig. S1a indicates the thickness of Te film is about 100 nm. As shown in Fig. S1b, the optical bandgap of the prepared Te (100 nm) is determined to be 0.37 ± 0.01 eV^1^. Additionally, the scanning electron microscopy (SEM) image clearly exhibits a continuous and uniform morphology.


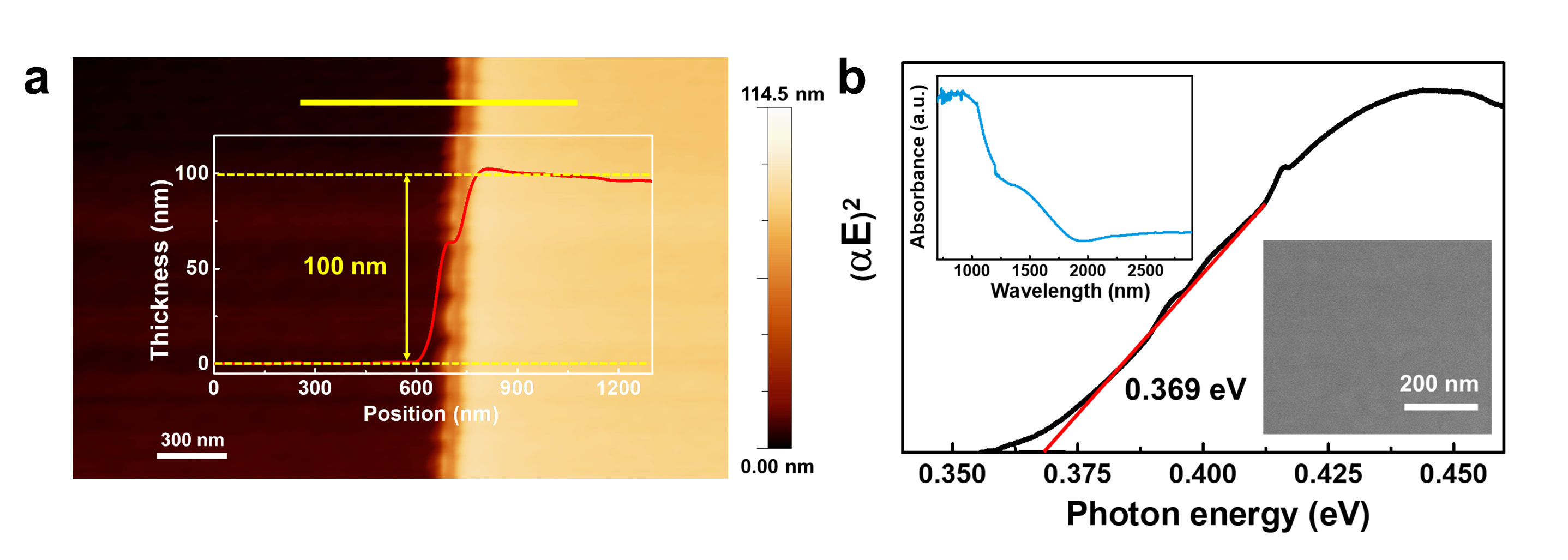


**Figure S1.** **Characterizations for Te.** **a** AFM image and the height profile along the marked yellow line. **b** Tauc plot versus photon energy. Insets are the absorption spectrum and the top-view SEM image.

**2. Fitting parameters of the biexponential function for the Te (100 nm)**

We have utilized the biexponential function $y=A_{0}{+A}_{1}e^{-\frac{x-x_{0}}{\tau_{1}}}+A_{2}e^{-\frac{x-x_{0}}{\tau_{2}}}$ with the added term *A*_0_, where *τ*_1_ and *τ*_2_ are relaxation times, *A*_1_ and *A*_2_ are the corresponding amplitudes, and *A*_0_ is the amplitude of transient component with long relaxation time that cannot be accurately determined from our data^2,3^. The fitting amplitude parameters are given in Table S1. The value of *A*_0_ is zero at the low pump fluence and rises with the increased fluence. Moreover, the small proportion of *A*_0_ compared to *A*_1_ and *A*_2_ implies that the contribution of *A*_0_ is weak in our observed transient processes.

**Table S1.** Fitting parameters of the biexponential function for Te (100 nm)

| Pump fluence (µJ cm^-2^) | 2.6 | 13 | 26 | 52 | 130 | 260 |
| --- | --- | --- | --- | --- | --- | --- |
| *A*_0_ | 0 | 0 | 0.5 | 1 | 1.4 | 2 |
| *A*_1_ | 5 | 10 | 16 | 20 | 25 | 30 |
| *A*_2_ | 10 | 23 | 33 | 40 | 52 | 65 |

**3. Experimental results of Te nanofilms**

To further study the influence of the pump wavelength on the relaxation process of Te, we measured the −Δ*T*/*T*_0_ under the 400 nm pump excitation shown in Fig. S2. The results indicate the *MD* reduces dramatically, probably due to the less absorption at 400 nm. The relaxation time constants given in the inset remain unchanged with the pump fluence and nearly the same as those with 800 nm pump.


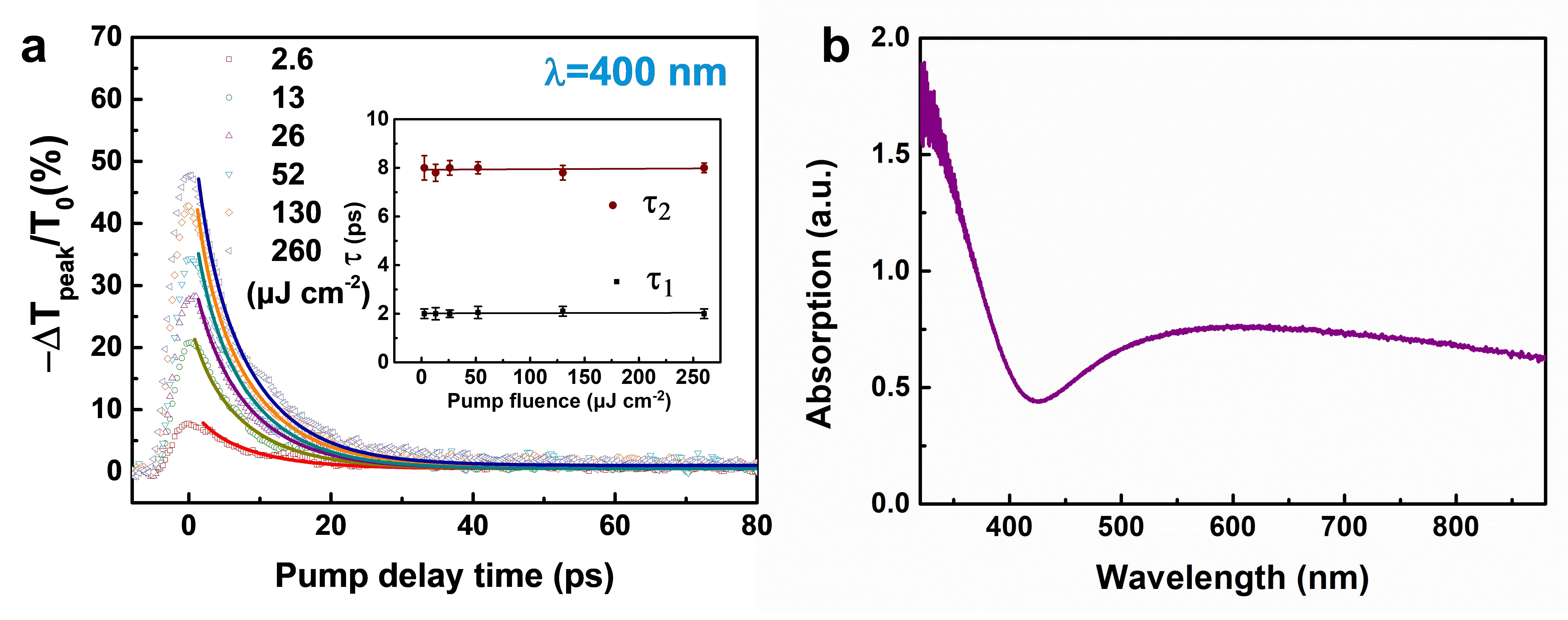


**Figure S2.** **Transient THz dynamics of Te (100 nm) under 400nm pump.** **a** −Δ*T*/*T*_0_ at the peak of THz amplitude with the various pump fluence at λ= 400 nm and **b** absorption spectra for Te (100 nm).


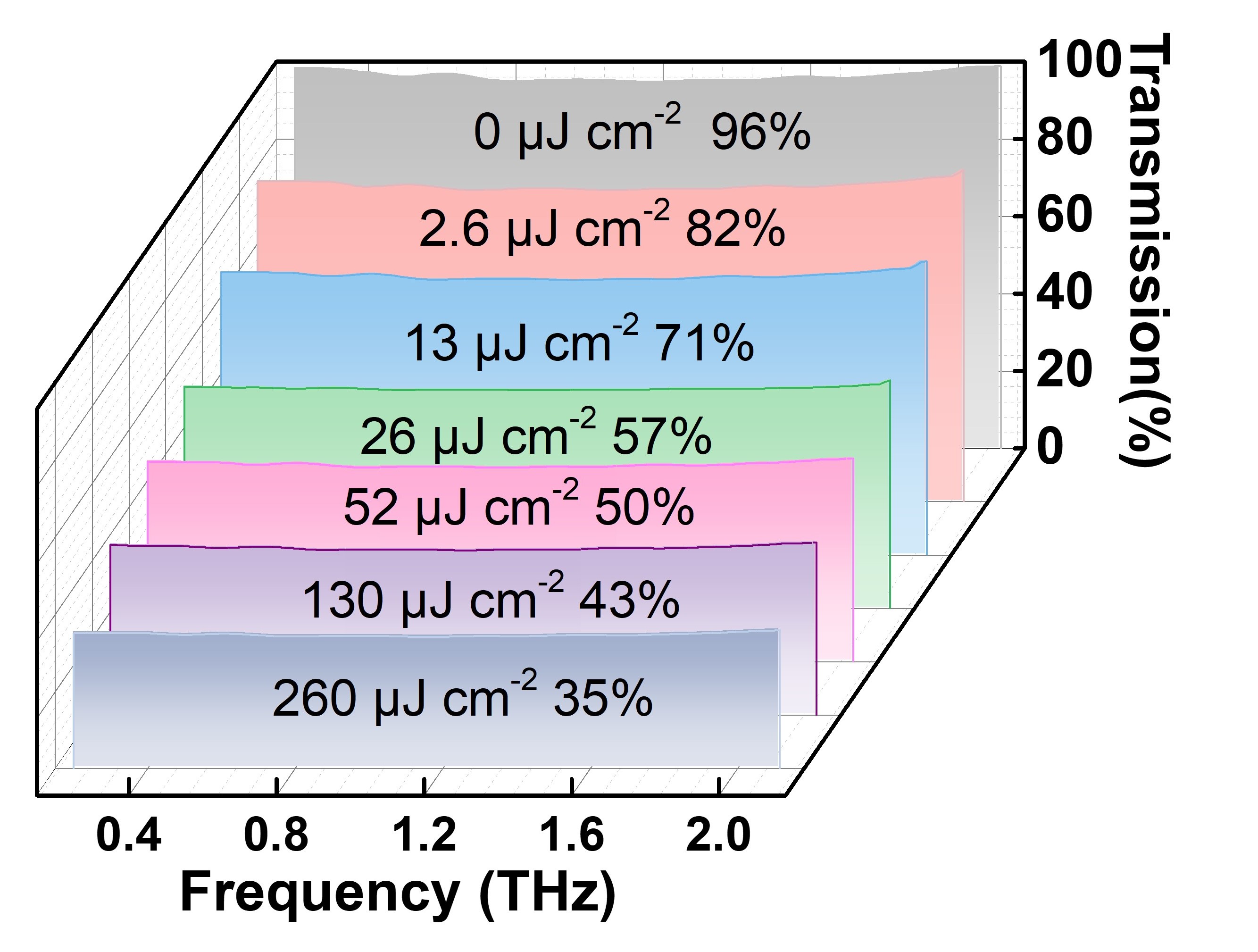


**Fig. S3.** Transmission spectra at pump delay time of 0 ps.

The transient photo-induced dynamics of Te nanofilms at pump fluence of 26 µJ cm^-2^ are presented in Fig. S4. Compared with the behaviors of Te (300 nm) and Te (50 nm), Te (100 nm) is the optimal candidate. The difference of optical properties in those films might be attributed to the defect states of the materials and the probability of the electron scattering dependent on the mean free path^4,5^.

Under the pump excitation, the conductivity of Te nanofilm will increase to induce the decrease in the skin depth of the incident THz waves, which can be observed from our measured results shown in Fig. S4c. For the Te nanofilms with the thicknesses of 50, 100, and 300 nm, respectively, their amplitude modulation depths exhibit obvious differences at low pump fluence, indicating that the skin depth of the pumped film is at least larger than 100 nm at the low conductivity. When the pump fluence is increased to 260 µJ cm^-2^, the modulation depths for Te (100 nm) and Te (300 nm) nanofilms are nearly the same, implying that the skin depth is around 100 nm. In addition, it is known that when the field propagates into the film, the amplitude decreases as *E*=*E*_0_e^-^*^αd^*, where *E*_0_ and *E* represent the amplitudes of incident and transmitted waves. *α* and *d* are the absorption coefficient and film thickness, respectively^6–8^. Then we calculate the amplitude skin depth *δ*=1/*α* with our experimental data for Te (100 nm), as shown in Fig. S4d. It is found that the skin depth of the pumped film at THz frequencies is remarkably reduced with the increasing pump fluence, showing the value is about 94 nm at 260 µJ cm^-2^. This could also confirm that the photoexcited Te film can suppress THz transmission.


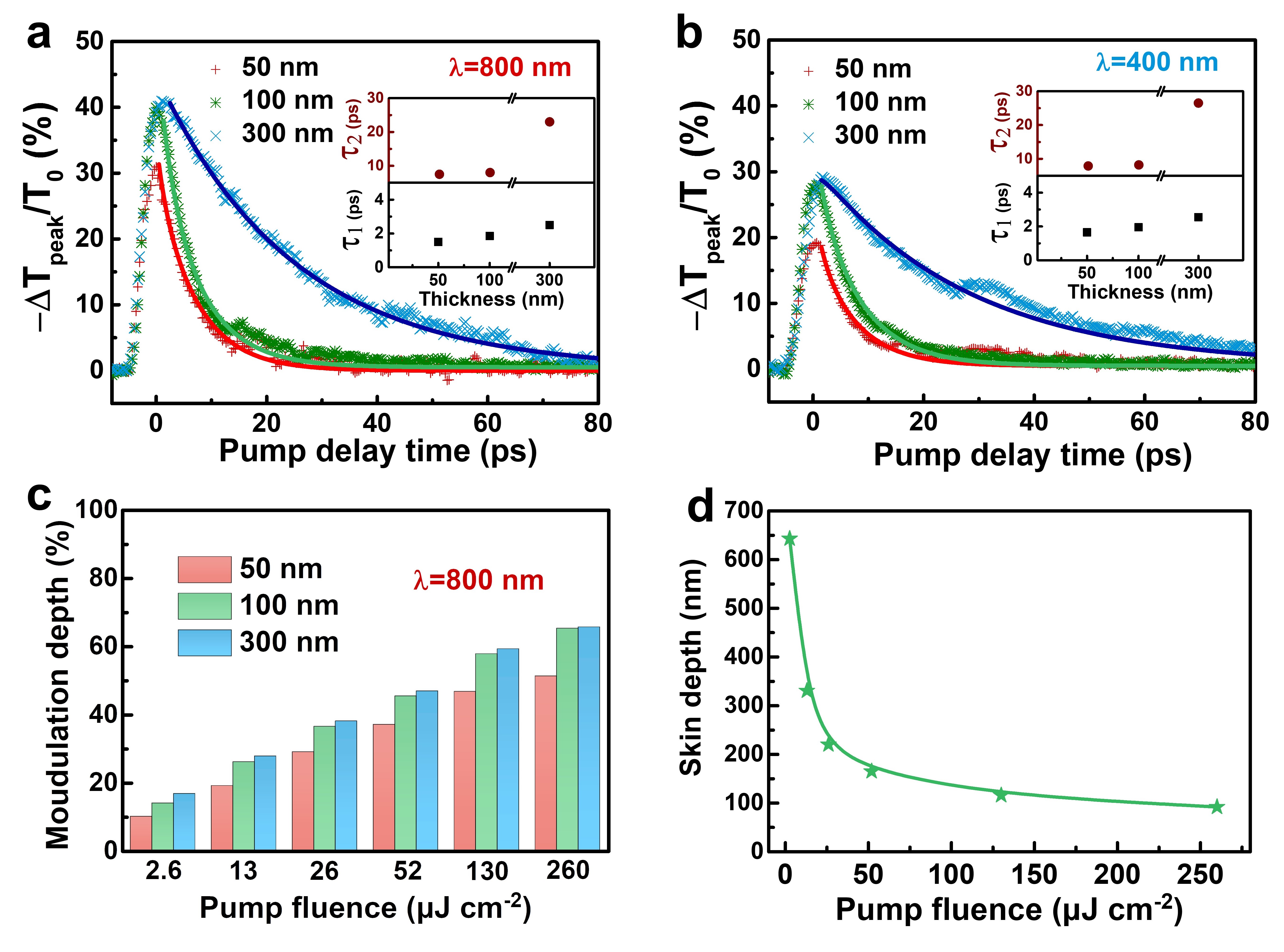


**Figure S4.** **Transient THz dynamics of Te with different thicknesses.** **a** −Δ*T*/*T*_0_ curves of Te nanofilms with different thickness at pump fluence of 26 µJ cm^-2^ under λ=800 nm and **b** λ=400 nm along with biexponential fitting, respectively. Insets: Pump fluence dependence of relaxation times (*τ*_1_ and *τ*_2_). **c** Modulation depth of Te under different pump fluence at 800 nm pump wavelength. **d** Calculated skin depth of Te (100 nm) film at THz frequencies under different pump fluence.

**Table S2.** Fitting parameters with the modified Drude-Smith model for Te (100 nm)

| Pump fluence (µJ cm^-2^) | 2.6 | 13 | 26 | 52 | 130 | 260 |
| --- | --- | --- | --- | --- | --- | --- |
| *N* (10^19^ cm^-3^) | 0.28 | 1.35 | 1.75 | 3.15 | 4.70 | 5.65 |
| *τ'* (fs) | 750 | 470 | 460 | 405 | 398 | 375 |
| *t_d_* (fs) | 21 | 25 | 29 | 36 | 45 | 53 |

**4. Zero crossing point of THz waveforms transmitted through Te**

The presence of excitons induces a phase shift in the THz pulse. Therefore, the exciton lifetime can be similarly determined by monitoring the light induced phase shift of THz transmission. In experiments, the phase shift can be quantified by measuring the change of the THz amplitude at a zero-crossing point, as shown in Fig. S5, through which one is able to exclude the contribution of the photoinduced amplitude change^9−11^.





**Figure S5.** Transmission of THz electric field (red solid line) and photomodulation of the pulse Δ*T* measured at 0 ps (blue solid curve) and 50 ps (green solid line) in the Te (100 nm).

**5. Fitting parameters of the biexponential function for the samples**

In Table S3, the time constants of the free carrier and exciton dynamics extracted by fitting the THz transient measured in Figs. 3a and 3c at peak position and zero crossing of THz pulse under pump fluence of 26 µJ cm^-2^.

**Table S3**. Fitting parameters of biexponential function under pump fluence of 26 µJ cm^-2^

| Charge species | *τ*(ps) | Gr | Te | Te/Gr | Gr/Te | Ge | Te/Ge | Ge/Te |
| --- | --- | --- | --- | --- | --- | --- | --- | --- |
| Free carrier | *τ*_1_ | 0.50±0.13 | 1.95±0.12 | 1.85±0.15 | 1.80±0.15 | 0.50±0.10 | 1.60±0.10 | 1.50±0.10 |
|  | *τ*_2_ | 1.60±0.15 | 8.00±0.25 | 7.80±0.20 | 7.90±0.25 | 1.55±0.20 | 7.70±0.20 | 7.75±0.25 |
| Exciton | *τ*_1_ | 0.50±0.10 | 0.60±0.15 | 0.60±0.20 | 0.30±0.15 | 0.20±0.11 | 0.60±0.10 | 0.30±0.15 |
|  | *τ*_2_ | 1.20±0.15 | 2.90±0.15 | 2.65±0.20 | 2.50±0.10 | 0.55±0.05 | 2.00±0.15 | 1.90±0.20 |

**6. Transient dynamics properties of Gr and Ge heterojunctions**

As given in Fig. S6, the THz transient dynamics measurements show that those heterojunctions formed by Gr with negative photoconductivity and Ge with positive photoconductivity only exhibit positive photoconductivity phenomena. In addition, the modulation depth at 26 µJ cm^-2^ is enhanced with the value of 9.8% for Ge/Gr and 10.7% for Gr/Ge with the ultrafast characteristics, indicating the stacking order has the influence on the modulation properties.


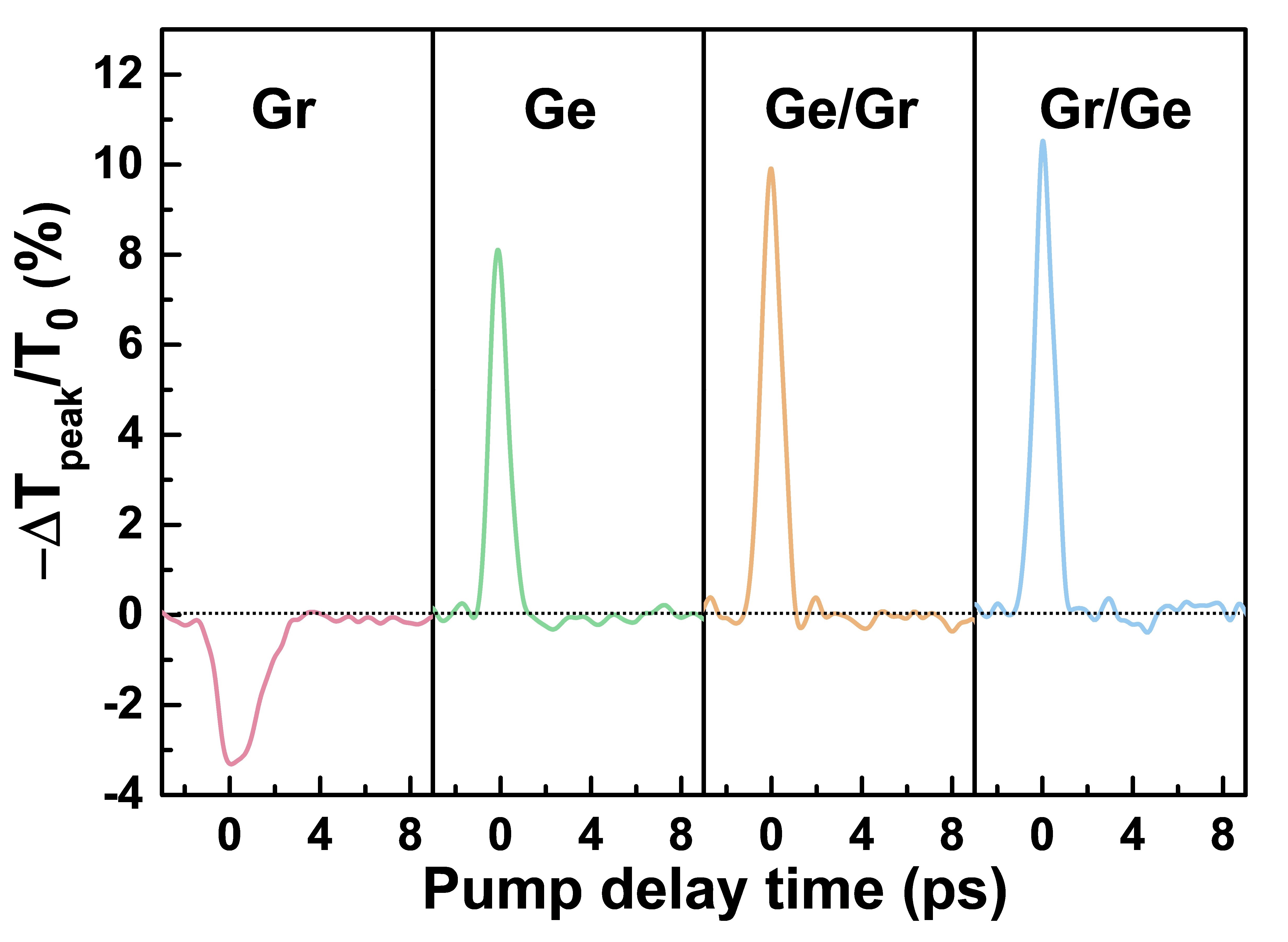


**Figure S6.** Transient dynamics properties −Δ*T*/*T*_0_ of Gr and Ge heterojunctions at the peak of THz amplitude under 800 nm pump with fluence of 26 µJ cm^-2^.

**7. DFT calculations**

The calculations based on DFT were performed by using the projected augmented wave (PAW) method as implemented in Vienna Ab Initio Simulation Package (VASP). A plane-wave cutoff energy of 450 eV was employed. The valence electron configurations of Te, Ge, Si, and O were 5*s*5*p*, 3*d*4*s*4*p*, 3*s*3*p*, and 2*s*2*p*, respectively. For the calculations of Ge and Te, the strongly constrained and appropriately normed (SCAN) functional was used to describe the exchange-correlation interactions for the structural optimizations. Basing on the relaxed structures, we applied hybrid Heyd-Scuseria-Ernzerhof (HSE) functional to calculate the electronic band structures since the electronic structure would be well described by using the hybrid DFT with spin orbit coupling (SOC). Meanwhile, a 5×5×5 *k*-points of Monkhorst-Pack mesh were used for Brillouin zone sampling. While for the calculations of Ge/Te layers on SiO_2_ substrate, the Perdew–Burke–Ernzerh (PBE) type generalized gradient approximation (GGA) was used for the exchange correlation potentials and a 3×3×1 *k*-points of Monkhorst-Pack meshes were applied. All the structures were relaxed until the Feynman–Hellman forces acting on each atom were less than 0.01 eV Å^-1^.

**8. Potential difference between Te and Ge in Te/Ge and Ge/Te heterojunctions**

The surface potential difference at Ge and Te interface is obtained by AFM measurement, as shown in Fig. S7. It is observed that Te has a higher Fermi energy level than Ge, providing the direct evidence of the existence of built-in electric fields. Moreover, different stacking order has a great influence on the potential difference with the value of only 3 mV in Te/Ge but 30 mV in Ge/Te due to the substrate effect.


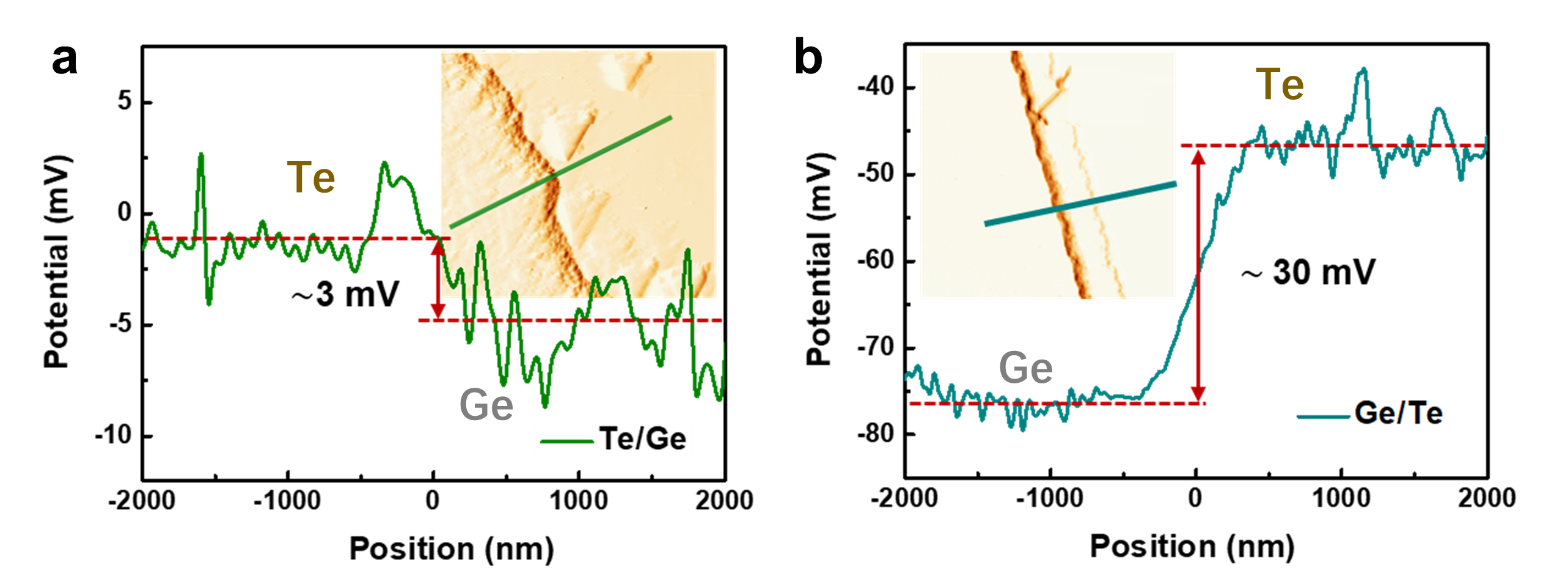


**Figure S7.** Potential difference between Te and Ge in Te/Ge and Ge/Te heterojunctions.

**References**

1. Dash, J. K., Chen, L., Dinolfo, P. H., Lu, T.-M. & Wang, G.-C. A Method Toward Fabricating Semiconducting 3R-NbS_2_ Ultrathin Films. *J. Phys. Chem. C* **119**, 19763–19771 (2015).

2. Li, G. *et al.* Dynamical Control over Terahertz Electromagnetic Interference Shielding with 2D Ti_3_C_2_T*_y_* MXene by Ultrafast Optical Pulses. *Nano Lett.* **20**, 636–643 (2020).

3. Srivastava, Y. K. *et al.* MoS_2_ for Ultrafast All‐Optical Switching and Modulation of THz Fano Metaphotonic Devices. *Adv. Opt. Mater.* **5**, 1700762 (2017).

4. Zhao, C. *et al.* Evaporated tellurium thin films for p-type field-effect transistors and circuits. *Nat. Nanotechnol.* **15**, 53–58 (2020).

5. Jin, Z. *et al.* Ultrafast electron transport in metallic antiferromagnetic Mn_2_Au thin films probed by terahertz spectroscopy. *Phys. Rev. B* **102**, 014438 (2020).

6. Ramo, S., Whinnery, J. R. & Van Duzer, T. Fields and waves in communications electronics. (Wiley, New York, 1994).

7.[Wentworth](https://ieeexplore.ieee.org/author/37355381400), S.M., [Wentworth](https://ieeexplore.ieee.org/author/37355381400), S. M., [Baginski](https://ieeexplore.ieee.org/author/37563599600), M. E., [Faircloth](https://ieeexplore.ieee.org/author/37562003900), D. L., [Rao](https://ieeexplore.ieee.org/author/37291303100), S. M. & [Riggs](https://ieeexplore.ieee.org/author/37360423600), L. S. Calculating effective skin depth for thin conductive sheets. In IEEE Antennas and Propagation Society International Symposium 4845-4848 (Albuquerque, NM, USA,2006).

8. Laman, N. & Grischkowsky, D. Terahertz conductivity of thin metal films. *Appl. Phys. Lett.* **93**, 051105 (2008).

9. He, C. *et al.* Competition between Free Carriers and Excitons Mediated by Defects Observed in Layered WSe_2_ Crystal with Time-Resolved Terahertz Spectroscopy. *Adv. Opt. Mater.* **6**, 1800290 (2018).

10. Xing, X. *et al.* Role of Photoinduced Exciton in the Transient Terahertz Conductivity of Few-Layer WS_2_ Laminate. *J. Phys. Chem. C* **121**, 20451–20457 (2017).

11. Lee, K. *et al.* Sub-Picosecond Carrier Dynamics Induced by Efficient Charge Transfer in MoTe_2_/WTe_2_ van der Waals Heterostructures. *ACS Nano* **13**, 9587–9594 (2019).
